# EBUS-TNBA 22G samples: Comparison of PD-L1 expression between DAKO and BIOCARE^®^

**DOI:** 10.7150/jca.35898

**Published:** 2019-08-20

**Authors:** Konstantinos Sapalidis, Paul Zarogoulidis, Dimitris Petridis, Christoforos Kosmidis, Barbara Fyntanidou, Kosmas Tsakiridis, Elena Maragouli, Aikaterini Amaniti, Dimitris Giannakidis, Charilaos Koulouris, Stylianos Mantalobas, Athanasios Katsaounis, Vyron Alexandrou, Georgios Koimtzis, Efstathios Pavlidis, Anastasios Barmpas, Theodora Tsiouda, Chrysanthi Sardeli, Zoi Aidoni, Haidong Huang, Qiang Li, Wolfgang Hohenforst-Schmidt, Isaak Kesisoglou

**Affiliations:** 1 3rd Department of Surgery, “AHEPA” University Hospital, Aristotle University of Thessaloniki, Medical School, Thessaloniki, Greece; 2 Department of Food Technology, School of Food Technology and Nutrition, Alexander Technological Educational Institute, Thessaloniki, Greece.; 3 Anesthisiology Department, “AHEPA” University Hospital, Aristotle University of Thessaloniki, Medical School, Thessaloniki, Greece; 4 Pulmonary Oncology Department, “Theageneio” Cancer Hospital, Thessaloniki, Greece; 5 Intensive Care Unit, “AHEPA” University Hospital, Aristotle University of Thessaloniki, Medical School, Thessaloniki, Greece; 6 Department of Pharmacology & Clinical Pharmacology, School of Medicine, Faculty of Health Sciences, Aristotle University of Thessaloniki, Thessaloniki, Greece; 7 The Diagnostic and Therapeutic Center of Respiratory Diseases, Shanghai East Hospital, Tongji University, Shanghai, China.; 8 Sana Clinic Group Franken, Department of Cardiology / Pulmonology / Intensive Care / Nephrology, "Hof" Clinics, University of Erlangen, Hof, Germany.

**Keywords:** NSCLC, PD-L1, convex probe EBUS, lymph nodes, lung cancer

## Abstract

**Introduction:** Lung cancer is diagnosed at advanced stage due to lack of early disease symptoms. Currently we have several different biopsy techniques such as; radial endobronchial ultrasound, convex probe endobronchial ultrasound, electromagnetic navigation, ct guided biospy and transthoracic ultrasound biopsy. Novel therapies such as; immunotherapy is being used for non-small cell lung cancer in the everyday clinical practice as first and second line treatment. Programmed ligand-1 is essential in order to administer immunotherapy as first line treatment.

**Patients and Methods:** Two thousands and two patients were included in our study where programmed ligand 1 was evaluated with DAKO technique and BIOCARE^®^. Cell blocks were obtain with convex probe ebus-tbna 22G needle.

**Results:** The Deming regression between DAKO and BIOCARE clone revealed an amazingly strong linear relationship as the coefficient of determination indicated (R^2^=0.999) and the variance ratio close to 1 (0.978), proving that both techniques can equally well be substituted for each other. The regression coefficient equals to 1 and the intercept hardly differs from 0 (0.936). In practice, this relationship permits adopting the economically affordable BIOCARE clone for further medical considerations.

**Conclusion:** No statistical difference was observed between DAKO and BIOCARE^®^, therefore we propose that both techniques can be used in order to investigate the expression of programmed ligand 1 with safety. PD-L1 expression was higher in the central mass instead of the lymphnodes.

## Introduction

Lung cancer is still diagnosed at a late stage due to lack of early symptoms. We are currently looking for an efficient proposal for lung cancer screening and early lung cancer detection^1^. There are certainly early results where an early detection program can reduce up to 20% of lung cancer deaths, however we still need to identify the population for screening and appropiate method 2. We have novel diagnostic equipment for periferal nodules or masses such as the radial ebus and electromagnetic navigation (EMN) 3-6. Moreover; cone beam ct is an additional equipment that we can use with both radial-ebus and EMN for a more efficient real time endobronchial navigation 7. Convex-Probe endobronchial ultrasound is used for the diagnosis of central masses/lymphadenopathy and lung cancer staging.^8^-^10^ It has been observed that the samples obtained from the needles are efficient for investigation of several genes.^11^-^13^ Epidermal growth factor (EGFR), anaplastic lymphoma kinase (ALK), ROS proto-oncogene 1 (ROS1), proto-oncogene B-Raf (BRAF) and programmed death-ligand 1 (PD-L1) can be investigated in the sample of a 21G, 22G and 19G needle that convex- probe EBUS is using 12. Cell-blocks are being created for 21 and 22G needles while this is not necessary for the 19G needle. We need cell-blocks or tissue to investigate ALK, ROS1, BRAF and PD-L1, while we need just cells or liquid biosy to investigate EGFR.^14^, ^15^ There are previously published studies where the PD-L1 can be investigated in cell-blocks 16-19. Currently the IHC 22C3pharmDx DAKO (Companion diagnostic system Dako, Denmark, EU) is validated and used in the everyday clinical practice for the evaluation of PD-L1 expression and in many countries the administration of immunotherapy is impossible if the expression has been determined with another clone 20. In the current study we investigated the expression of PD-L1 in NSCLC stage IIIB and IV patients with two clones in the same cell-block the IHC 22C3pharmDx DAKO (Companion diagnostic system Dako, Denmark, EU) and [clone: CAL10 (RTU, CE, IVD), Biocare, CA, USA). The primary endpoint was to investigate whether there would be a difference between the expression of the two clones and the secondary endpoint to investigate the expression of PD-L1 between the main site (mass) and metastatic lymphnodes.

## Patients and Methods

### Patients

**Figure 1:**
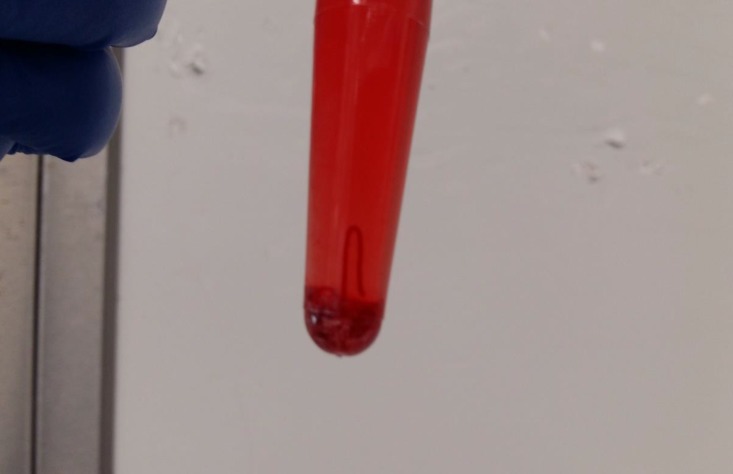
Sample material with 22G needle

In total two housand and two patients were included from five different departments from the 1st November 2013 to 31st March 2019. In our retrospective study we included patients that were eligible for diagnosis specifically with the convex-probe EBUS procedure and 22G needle. **Figure 1.**

The patients included were either Stage IIIb or Stage IV (supplementary data). In specific all patients had enlarged lymphnodes of the mesothorax that could be punctured with convex-probe EBUS (stations 2R/L, 4L/R, 7, 10L/R, 11L and 11superior and inferior). Some of the patients had also a central mass where it could be punctured with the operating system. None of the patients had an endobronchial mass. Therefore there were 3 group of patients; a) only mass, b) only lymph nodes and c) both sites. In any case all patients were stage IIIB or stage IV. (**Table 1.**) The histology types included were NSCLC if it was impossible to subcategorize to adenocarcinoma or squamus, adenocarcinoma, squamous cell carcinoma and mixed (adenocarcinoma and squamous cell carcinoma).

### Methods

**Figure 2:**
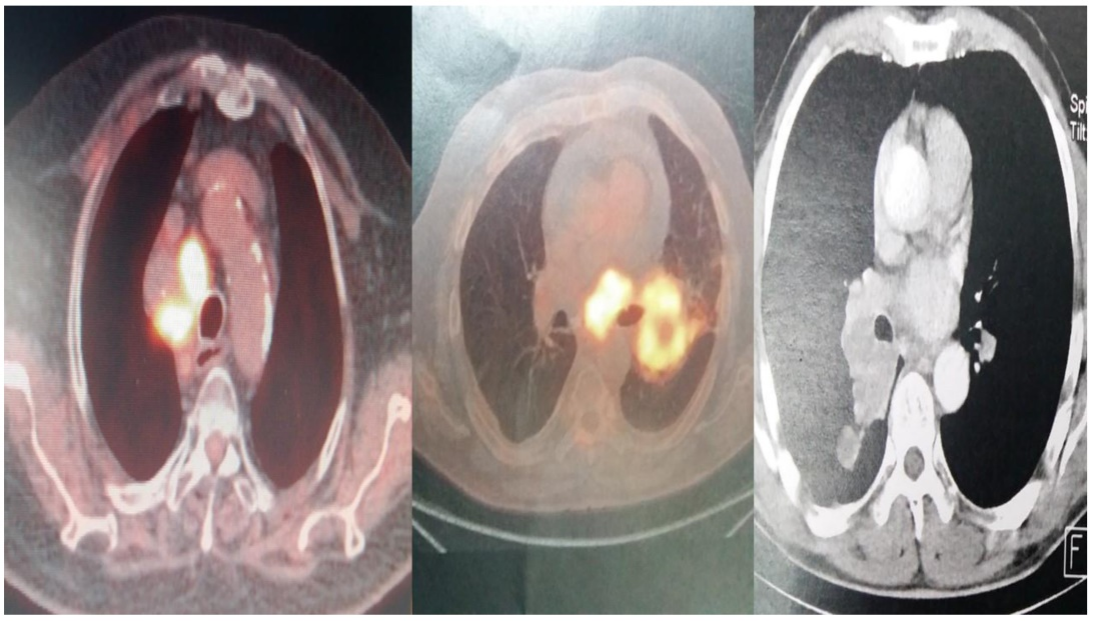
PET-CT and CT scan of different patients included

**Figure 3:**
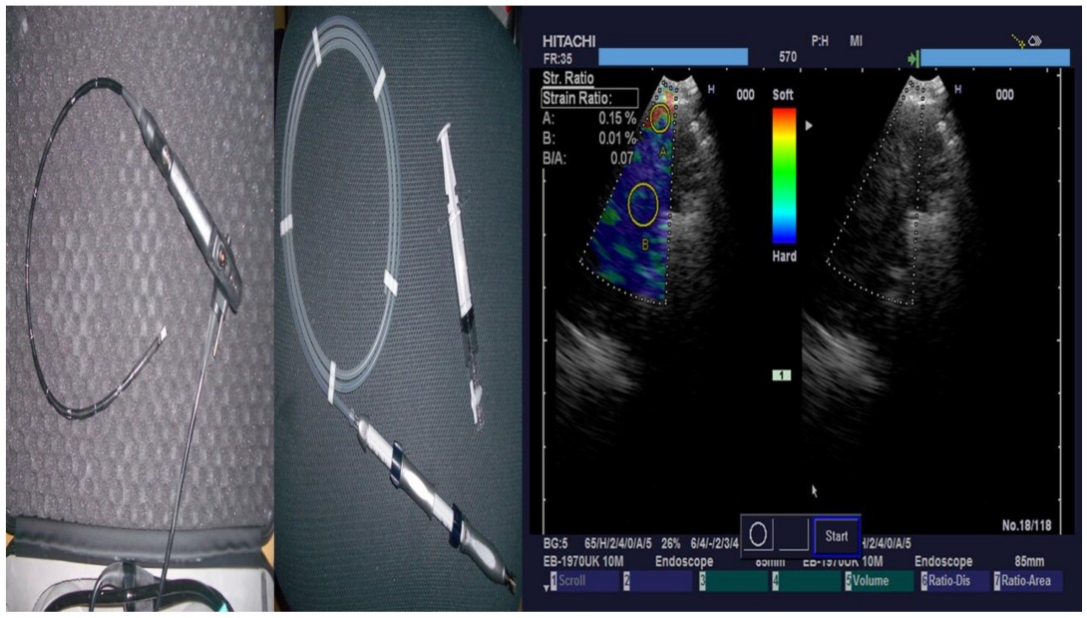
From left to right; PENTAX Convex-Probe EBUS, 22G Needle and right elastography during procedure (blue being the optimal biopsy site)

The convex-probe EBUS PENTAX EB-1970UK with a 22G needle was used to take biopsies from patients either from the main site, metastatic lymphnodes or both. All patients were sedated and a rigd bronchoscope (STORZ 12mm with a 11mm working channel) or tracheal tube number 8.5 or 9 was instered. The mean procedure time from intubation to last biopsy (four in total for every site) was 15 minutes. In the operating theater there was the operator, a nurse and the anesthisiologist. In all patients jet-ventilation respiration mode was used in order to avoid hypercapnia. All patients had previously CT of the thorax, PET-CT and elastography was used during the procedure in order to evaluate the pancture site (**Figures 2-3**).

### Cell-Blocks

**Figure 4:**
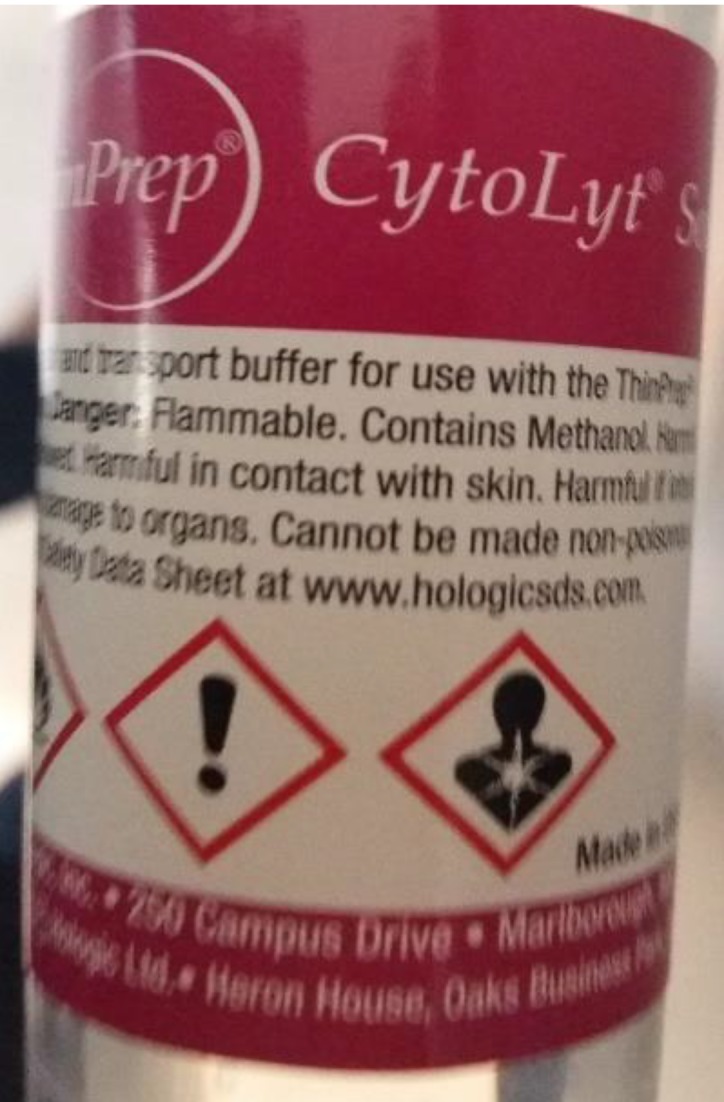
Storage bottle of the biopsy material

The speciment of fine needle aspiration (FNA) remains in the hub of cytolyt solution until it arrives in the laboratory. The material centriguged in a centrifuge tube at 2000rpm for ten minutes. Microscopic tissue fragments are easily recoverable in paraffin cellblock and it follows the protocol of a tissue device in the histopathology laboratory. This process during 10h41min. Routine H&E staining is used on all cellblock sections. (**Figure 4.**)

### PD-L1 evaluation

#### Staining protocol for PD-L1 IHC 22C3pharmDx DAKO (Companion diagnostic system Dako,Denmark,EU.)

**Figure 5:**
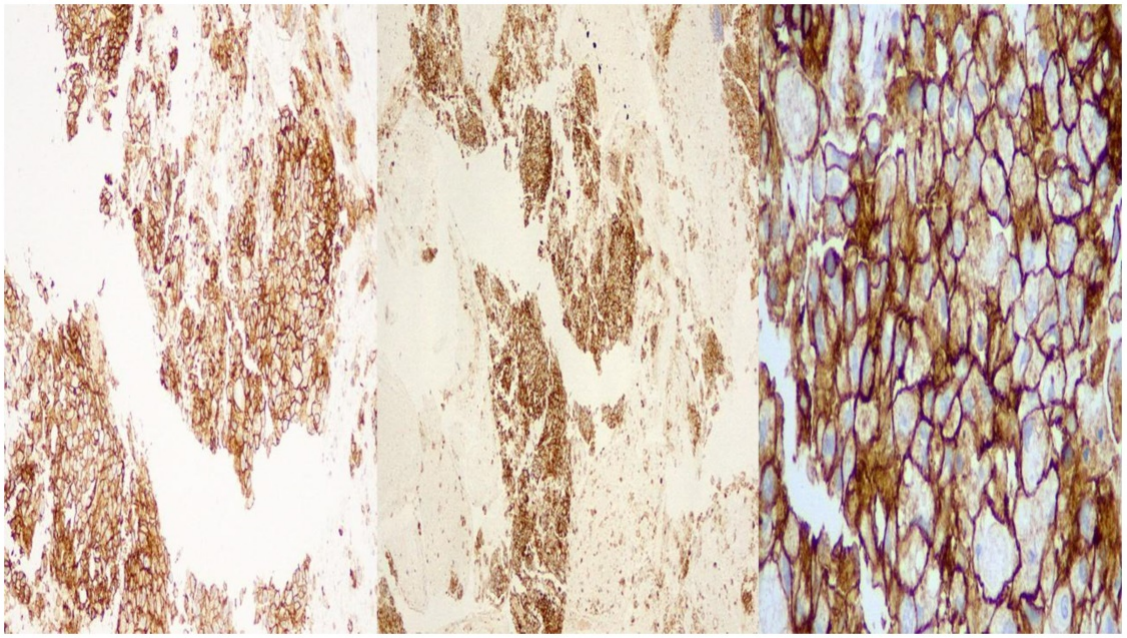
DAKO: left side X 10 magnification, middle: X 40 magnification, right side: X 400 magnification

Tissue sections of 2μ wide were performed on positive charged slides and deparafinized in an incubator at 56^0^ C. Dehydration of the sections followed and the tissues embedded in Target Retrieval Solution, Low pH (Code RT100/PT101/PT200 Dako) for 20 minutes at 66o C for antigen retrieval. When the tissues retained room temperature, were capable for the automate technique in the Autostainer Link 48 (Dako) under complete automate and stable conditions using additionally external positive and negative cell line controls. Primary and secondary antibodies performed and followed by polymer, Link reagents and Counterstain with Hematoxyline (CodeK8008). Rinsing and reagents time were preprogrammed in the Dako Link software. Mounting of the sections were performed outside of the Autostainer with non-aqueous, permanent media. (**Figure 5.**)

#### Staining protocol for Programmed death-ligand 1 (PD-L1) [clone: CAL10 (RTU, CE, IVD), Biocare, CA, USA)

**Figure 6:**
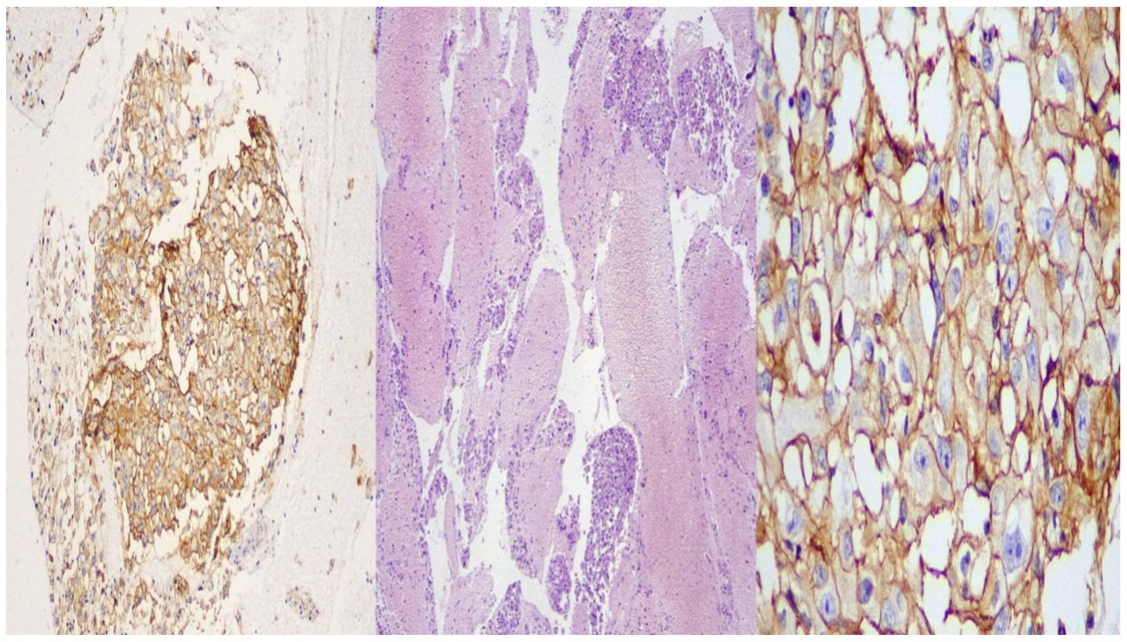
BioCare: left side X 10 magnification, middle: X 40 magnification, right side: X 400 magnification.

Immunohistochemistry was performed in 2-micron tissues on positive charged slides after deparafinization, dehydration and antigen retrieval which were completed in the Autostainer platform BOND MaxTM (Leica Biosystems, Wetzlar,G). The primary antibody ((PD-L1) [clone: CAL10 (RTU, CE, IVD), Biocare, CA, USA) was “ready to use” (RTU) and the incubation time was 20 minutes followed by secondary antibody and Linker, provided by the Autostainer platform reagents. Then the sections were counterstained with Hematoxylin and mounted with non-aqueous, permanent media. For external positive control, tonsil sections were used. (**Figure 6.**)

## Results

### Statistical analysis

The two testing techniques DAKO and BIOCARE were recorded quantitatively as expressions 2(DAKO technique) and 3(BIOCARE technique) correspondingly. A Deming regression was applied to the two expressions in order to explore how closely are affiliated each other. This regression also known as Model 2 assumes that both variables are measured randomly and thus share their own variation in the relationship. Data were analyzed using the JMP 14.0 (2018, SAS Institute Inc.) software.

### Results

**Figure 7:**
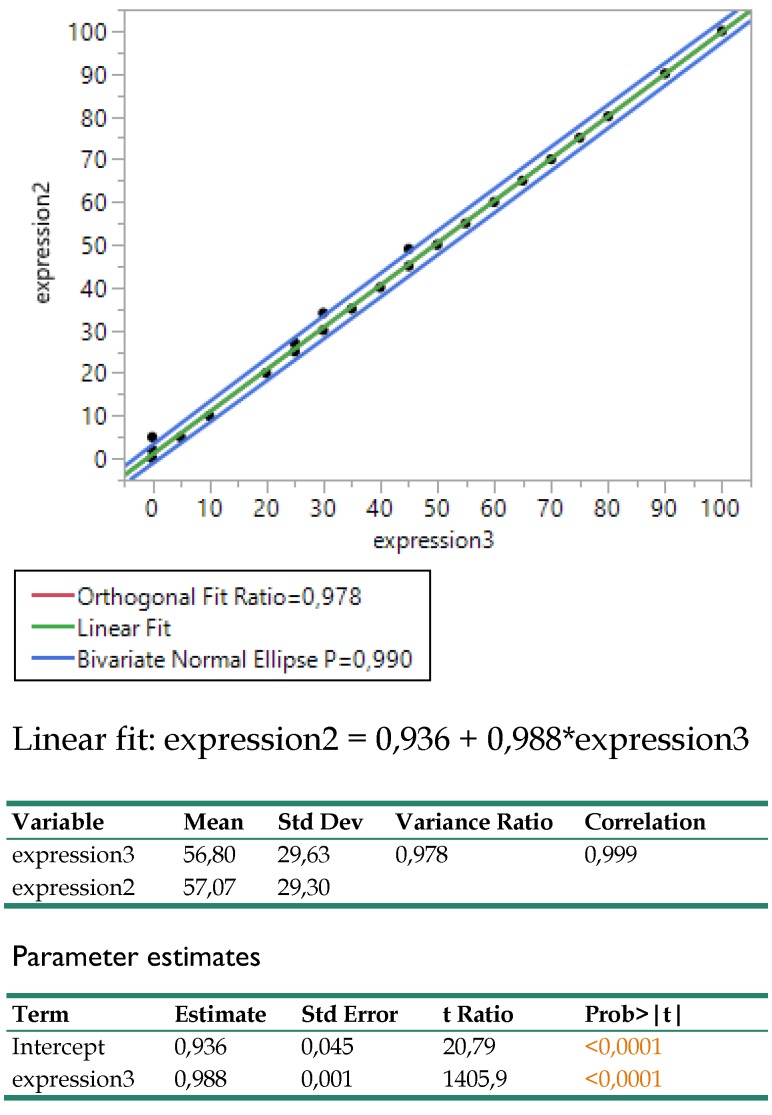
Bivariate regression between the expressions 2 (DAKO) and 3 (BIOCARE) and related statistics.

The Deming regression between expressions 2 (DAKO technique) and 3 (BIOCARE technique) revealed an amazingly strong linear relationship (**Figure 7.**) as the coefficient of determination indicated (R^2^=0.999) and the variance ratio close to 1 (0.978), proving that both techniques can equally well be substituted for each other. The regression coefficient equals to 1 and the intercept hardly differs from 0 (0.936). In practice, this relationship permits adopting the economically affordable expression3 (BIOCARE technique) for further medical considerations.

The traditionally applied expression2(DAKO technique) was further regressed against infection site, meta locations, histology type and PY to detect potential effects on response. The results are shown in **Table 2.** in which site, meta and histology (in order of significance) explain 44.1% of the total variation excluding the insignificant effect of PY.

The infection site signals that areas 0 (mass) and 2 (both mass and lymphnodes) attain equally (confidence intervals of means overlap) much higher expression values than that in site 1 (lymph nodes) (**Table 3.**).

Meta locations show clearly two patterns of expression means, one with low values composed by the categories 1 (bone), 2 (BRAIN) and 3 (lymph nodes) and another with high values including the locations 4 (liver), 5 (adrenal gland) and 7 (more than 1 metastasis). (**Table 4**.)

Interestingly, the expression means of meta locations 1 (bones) and 2 (brain) are significantly below the threshold of 50 units since their confidence intervals do not cross that value.

Finally, histology type 1 (adenocarcinoma) reaches an expression mean value greater than 70 units which is obviously much higher than those from the types 0 (NSCLC) and 2 (squamous cell carcinoma) whose means also do not differ significantly. (**Table 5.**)

## Discussion

The investigation of PD-L1 expression in patients with advanced stage disease is crucial. Previously published studies in NSCLC with immunotherapy as first line treatment alone have presented increased progression free survial and longlasting responses in specific patients with increased PD-L1 expression 21, 22. Currently there is an investigation towards combining chemotherapy and/or radiotherapy in order to increase the immunotherapy treating effect as first line treatment 23, 24. In any case the first step is to have a representative tissue sample from the disease and then make a molecular analysis. The word “representative” corresponds to the sample from the primary site, where tumor burden is higher and it is most likely that all mutations if any exist in a patient and can be identified. Based on previous studies and our current study the expression of PD-L1 was higher in the main site instead of lymphnodes 25. It has been observed during surgery that metastatic lymphnodes had less PD-L1 expression compared to the primary site (mass), an observation that we also made 26. There was no difference of PD-L1 expression between the two sex, although there is recent literature where the sex is a predictive disease marker for immunotherapy 27. There is a previously published study were small biopsies were identified to be as reliable as surgical biopsies 26. We have observed PD-L1 expression heterogeneity in tumors other than lung cancer, however; one could suspect the same for lung cancer 28, 29. There are previous studies where regarding EGFR mutations we concluded that “tissue” is the issue, meaning that we might have acquired a sample from the tumor however not a part with PD-L1 expression or ≥50 PD-L1 expression which is the cut off measurement for first line single agent immunotherapy 30. There are patients which very rarely present two mutations such as, EGFR and PD-L1, even in the case where PD-L1 expression is ≥50 then the patient should be treated with tyrosine kinase inhibitor 31. Tumor molecular transformation has been also observed where patients without PD-L1 expression after surgery presented PD-L1 expression 13. Elastography is a method for rapid on site evaluation of a lesion in order to investigate benignancy or malignancy. Moreover, we can evaluate the best site to puncture and acquire sample, although to target a specific area is very difficult in lesions ≤3cm 32, 33. In our current study we present a large number of patients with cell-block biopsies which we know from previous studies that is an efficient material for PD-L1 expression investigation. We observed that both methods have the same measurements with and the slightest differences we conclude that they were due to the difference slice obtained from the cell block in order to do the measurement of the second clone. In our study we concluded that PD-L1 expression is higher in the primary site instead of lymphnode in the mesothorax, however; we do not have data for other metastatic sites. To date only clinical observation during treatment with immunotherapy can justify a biopsy in an non-responding metastatic site. Indeed we have observed in case reports that non-responding metastatic do not have molecular expression of the primary site (EGFR, ALK, ROS1, ALK or PD-L1) or there are cases where we have a different lung cancer or lung cancer transformation 34-36.

## Figures and Tables

**Table 1:** Patients characteristics

	Male	Female
**Mass**	309	149
**Lymph Nodes**	188	393
**Both sites**	368	495
**Stage IIIB**	152	197
**Stage IV**	813	840
**Mean Age**	60.8	60
**Mean PY**	60.5	60.2

**Mass:** biopsy only at the tumor; **Lymph nodes:** biopsy only in lymph nodes next to the tumor site; **Both:** biopsy in central mass and lymphnodes

**Table 2:**
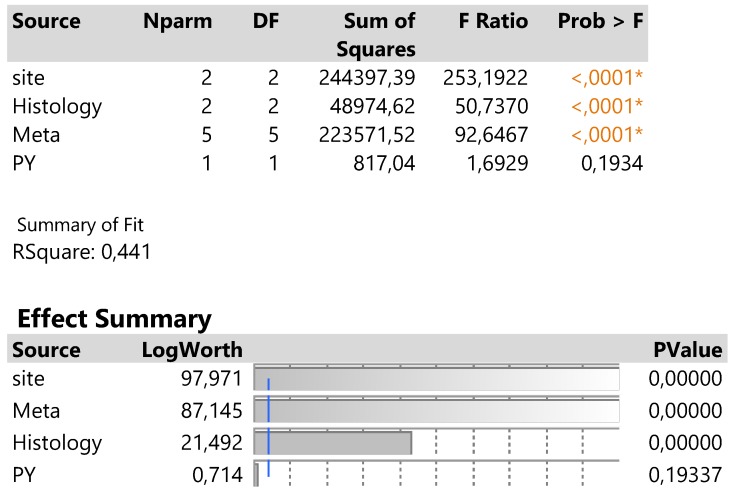
Analysis of variance of four main effects on the expression2 response and order of significance based on the logworth exact probability value, -log(p).

**Table 3:**
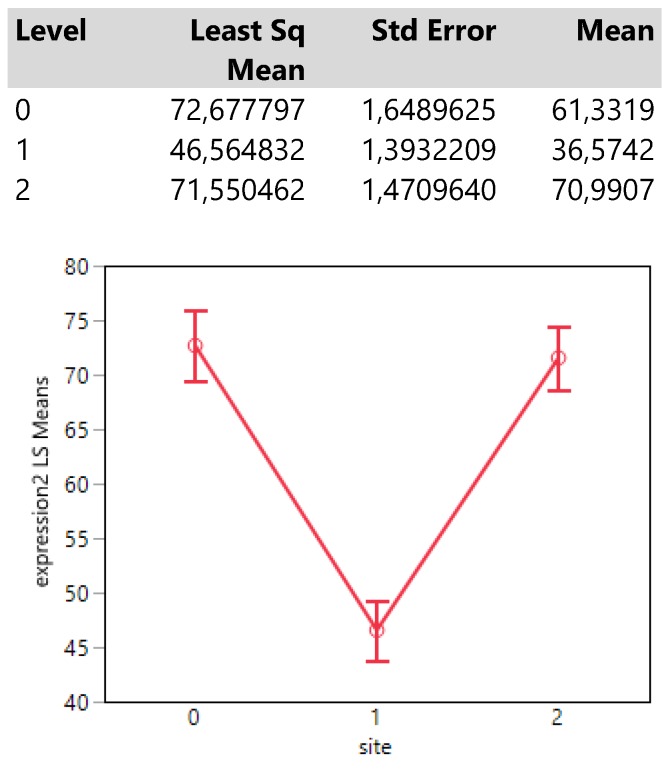
Descriptive statistics and least square means plot of expression2 in the three infection sites.

**Table 4:**
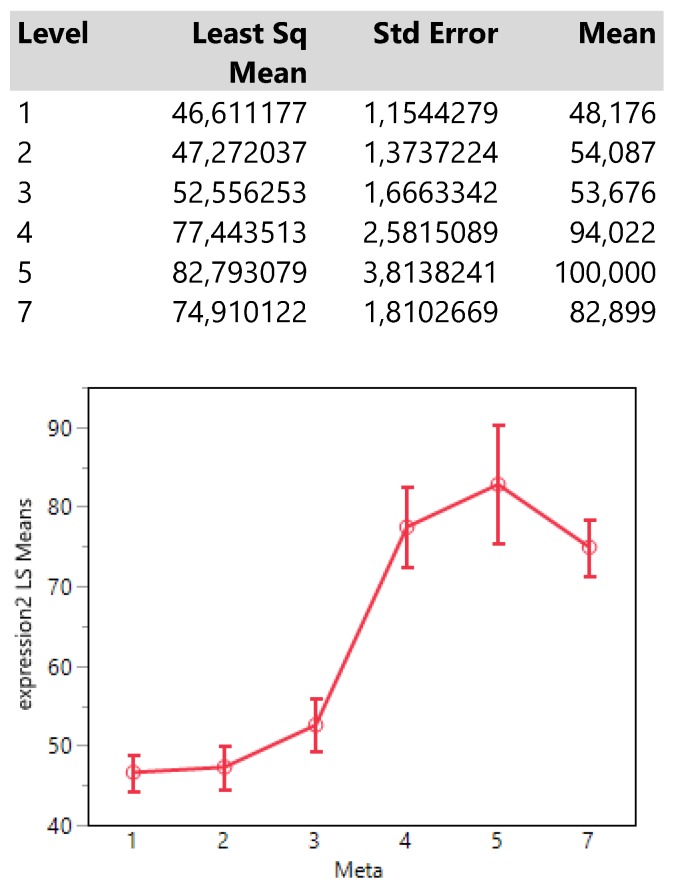
Descriptive statistics and least square means plot of expression2(DAKO technique) in the seven meta locations.

**Table 5:**
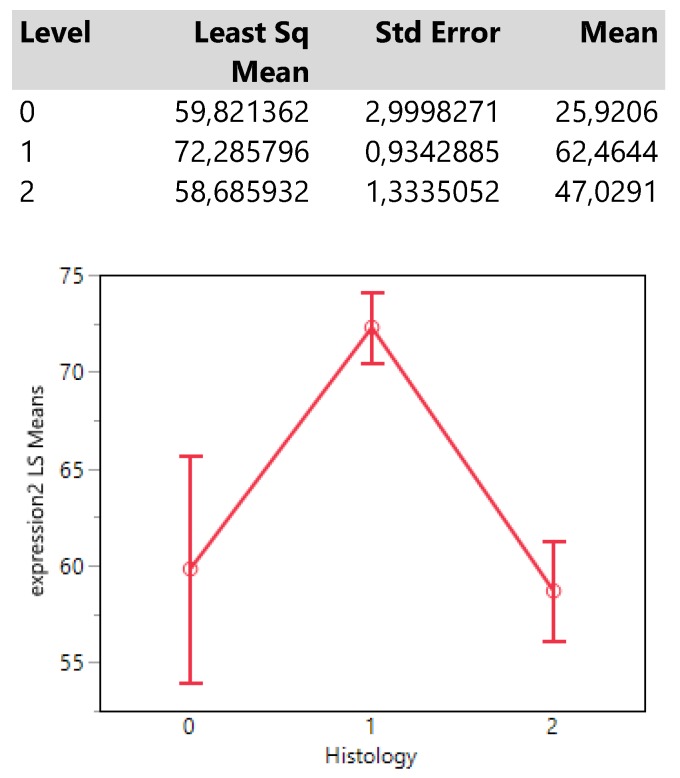
Descriptive statistics and least square means plot of expression2 (DAKO technique) in the three histology types.

## References

[1] Oudkerk M, Devaraj A, Vliegenthart R, Henzler T, Prosch H, Heussel CP (2017). European position statement on lung cancer screening. The Lancet Oncology.

[2] Nanavaty P, Alvarez MS, Alberts WM (2014). Lung cancer screening: advantages, controversies, and applications. Cancer control: journal of the Moffitt Cancer Center.

[3] Huang Z, Huang H, Ning Y, Han J, Shen Y, Shi H (2019). Radial probe endobronchial ultrasound assisted conventional transbronchial needle aspiration in the diagnosis of solitary peribronchial pulmonary lesion located in the segmental bronchi. Journal of Cancer.

[4] Haidong H, Yunye N, Wei Z, Zarogoulidis P, Hohenforst-Schmidt W, Man YG (2017). Multiple guided technologies based on radial probe endobronchial ultrasound for the diagnosis of solitary peripheral pulmonary lesions: a single-center study. Journal of Cancer.

[5] Zaric B, Stojsic V, Carapic V, Kovacevic T, Stojanovic G, Panjkovic M (2016). Radial Endobronchial Ultrasound (EBUS) Guided Suction Catheter-Biopsy in Histological Diagnosis of Peripheral Pulmonary Lesions. Journal of Cancer.

[6] Zaric B, Stojsic V, Sarcev T, Stojanovic G, Carapic V, Perin B (2013). Advanced bronchoscopic techniques in diagnosis and staging of lung cancer. Journal of thoracic disease.

[7] Hohenforst-Schmidt W, Zarogoulidis P, Vogl T, Turner JF, Browning R, Linsmeier B (2014). Cone Beam Computertomography (CBCT) in Interventional Chest Medicine - High Feasibility for Endobronchial Realtime Navigation. Journal of Cancer.

[8] Zarogoulidis P, Huang H, Bai C, Kosmidis C, Trakada G, Veletza L (2017). Endobronchial ultrasound convex probe for lymphoma, sarcoidosis, lung cancer and other thoracic entities. A case series. Respiratory medicine case reports.

[9] Huang H, Huang Z, Wang Q, Wang X, Dong Y, Zhang W (2017). Effectiveness of the Benign and Malignant Diagnosis of Mediastinal and Hilar Lymph Nodes by Endobronchial Ultrasound Elastography. Journal of Cancer.

[10] Zarogoulidis P, Laskou S, Katsaounis A, Pavlidis E, Giannakidis D, Koulouris C (2018). Esophagus lyomyoma diagnosed with convex endobronchial ultrasound (EBUS). Respiratory medicine case reports.

[11] Yarmus L, Akulian J, Gilbert C, Feller-Kopman D, Lee HJ, Zarogoulidis P (2013). Optimizing endobronchial ultrasound for molecular analysis. How many passes are needed?. Annals of the American Thoracic Society.

[12] Oezkan F, Khan A, Zarogoulidis P, Hohenforst-Schmidt W, Theegarten D, Yasufuku K (2014). Efficient utilization of EBUS-TBNA samples for both diagnosis and molecular analyses. OncoTargets and therapy.

[13] Kosmidis C, Koimtzis G, Giannakidis D, Tteralli N, Mantalovas S, Tsakalidis A (2019). Transformation of ALK expression and PD-L1 0% to PD-L1 90% only after surgery: the need for rebiopsy in lung cancer patients. International medical case reports journal.

[14] Tsoulos N, Papadopoulou E, Metaxa-Mariatou V, Tsaousis G, Efstathiadou C, Tounta G (2017). Tumor molecular profiling of NSCLC patients using next generation sequencing. Oncology reports.

[15] Zhang YC, Zhou Q, Wu YL (2017). The emerging roles of NGS-based liquid biopsy in non-small cell lung cancer. Journal of hematology & oncology.

[16] Xu H, Bratton L, Nead M, Russell D, Zhou Z (2018). Comparison of programmed death-ligand 1 (PD-L1) immunostain for nonsmall cell lung carcinoma between paired cytological and surgical specimens. CytoJournal.

[17] Hernandez A, Brandler TC, Zhou F, Moreira AL, Schatz-Siemers N, Simsir A (2019). Assessment of Programmed Death-Ligand 1 (PD-L1) Immunohistochemical Expression on Cytology Specimens in Non-Small Cell Lung Carcinoma. American journal of clinical pathology.

[18] Wang H, Agulnik J, Kasymjanova G, Wang A, Jimenez P, Cohen V (2018). Cytology cell blocks are suitable for immunohistochemical testing for PD-L1 in lung cancer. Annals of oncology: official journal of the European Society for Medical Oncology.

[19] Noll B, Wang WL, Gong Y, Zhao J, Kalhor N, Prieto V (2018). Programmed death ligand 1 testing in non-small cell lung carcinoma cytology cell block and aspirate smear preparations. Cancer cytopathology.

[20] Udall M, Rizzo M, Kenny J, Doherty J, Dahm S, Robbins P (2018). PD-L1 diagnostic tests: a systematic literature review of scoring algorithms and test-validation metrics. Diagnostic pathology.

[21] Peters S, Kerr KM, Stahel R (2018). PD-1 blockade in advanced NSCLC: A focus on pembrolizumab. Cancer treatment reviews.

[22] Smit EF, de Langen AJ (2019). Pembrolizumab for all PD-L1-positive NSCLC.

[23] Maity A, Mick R, Huang AC, George SM, Farwell MD, Lukens JN (2018). A phase I trial of pembrolizumab with hypofractionated radiotherapy in patients with metastatic solid tumours. British journal of cancer.

[24] Langer CJ, Gadgeel SM, Borghaei H, Papadimitrakopoulou VA, Patnaik A, Powell SF (2016). Carboplatin and pemetrexed with or without pembrolizumab for advanced, non-squamous non-small-cell lung cancer: a randomised, phase 2 cohort of the open-label KEYNOTE-021 study. The Lancet Oncology.

[25] Aguiar PN Jr, De Mello RA, Hall P, Tadokoro H, Lima Lopes G (2017). PD-L1 expression as a predictive biomarker in advanced non-small-cell lung cancer: updated survival data. Immunotherapy.

[26] Kim I, Kim A, Lee CH, Lee G, Kim A, Jo EJ (2019). Reliability of PD-L1 assays using small tissue samples compared with surgical specimens. Medicine.

[27] Wang S, Zhang J, He Z, Wu K, Liu XS (2019). The predictive power of tumor mutational burden in lung cancer immunotherapy response is influenced by patients' sex. International journal of cancer.

[28] Rasmussen JH, Lelkaitis G, Hakansson K, Vogelius IR, Johannesen HH, Fischer BM (2019). Intratumor heterogeneity of PD-L1 expression in head and neck squamous cell carcinoma.

[29] Zarogoulidis P, Papadopoulos V, Maragouli E, Papatsibas G, Karapantzos I, Bai C (2018). Tumor heterogenicity: multiple needle biopsies from different lesion sites-key to successful targeted therapy and immunotherapy. Translational lung cancer research.

[30] Zarogoulidis P, Gaga M, Huang H, Darwiche K, Rapti A, Hohenforst-Schmidt W (2017). Tissue is the issue and tissue competition. Re-biopsy for mutation T790: where and why?. Clinical and translational medicine.

[31] Zarogoulidis P, Chinelis P, Efthymiou C, Athanasiadou A, Mpikos V, Papatsibas G (2017). EGFR or PD-L1 decision for first line therapy in a case series of EGFR positive and PD-L1 >50. Respiratory medicine case reports.

[32] Shiina Y, Nakajima T, Suzuki H, Yoshino I (2019). Localization of the Metastatic Site Within a Lymph Node Using Endobronchial Elastography. Seminars in thoracic and cardiovascular surgery.

[33] Rozman A, Malovrh MM, Adamic K, Subic T, Kovac V, Flezar M (2015). Endobronchial ultrasound elastography strain ratio for mediastinal lymph node diagnosis. Radiology and oncology.

[34] Le T, Sailors J, Oliver DH, Mayer M, Hoskin S, Gerber DE (2017). Histologic transformation of EGFR mutant lung adenocarcinoma without exposure to EGFR inhibition. Lung cancer.

[35] Gong J, Gregg JP, Ma W, Yoneda K, Moore EH, Daly ME (2019). Squamous Cell Transformation of Primary Lung Adenocarcinoma in a Patient With EML4-ALK Fusion Variant 5 Refractory to ALK Inhibitors. Journal of the National Comprehensive Cancer Network: JNCCN.

[36] Yang H, Liu L, Zhou C, Xiong Y, Hu Y, Yang N (2019). The clinicopathologic of pulmonary adenocarcinoma transformation to small cell lung cancer. Medicine.

